# Cost-effectiveness of the treatment of traumatic thoracolumbar spine fractures: Nonsurgical or surgical therapy?

**DOI:** 10.4103/0019-5413.36997

**Published:** 2007

**Authors:** Jan Siebenga, Michiel JM Segers, Vincent JM Leferink, Matthijs J Elzinga, Fred C Bakker, Henk-Jan ten Duis, Pol M Rommens, Peter Patka

**Affiliations:** Atrium Medisch Centrum Parkstad, Heerlen, the Netherlands; * Vrije Universiteit Medisch Centrum, Amsterdam, Netherlands; ** Academisch Ziekenhuis Groningen, Groningen, the Netherlands; *** Universitätsklinik Mainz, Mainz, Germany; **** Erasmus University Rotterdam, The Netherlands

**Keywords:** Cost-effectiveness, RCT, thoracolumbar fracture, treatment

## Abstract

**Background:**

Spinal fractures can be an important cause for disabling back pain. Therefore, in judging the cost-effectiveness of nonsurgical or surgical therapy, not only direct costs but also the indirect costs should be calculated. In this prospective randomized study, the costs incurred by nonsurgically and surgically treated patients with a traumatic thoracolumbar spine fracture without neurological involvement were analysed.

**Materials and Methods:**

32 patients with a traumatic thoracolumbar spine fracture were prospectively randomized for operative or nonsurgical treatment. Patients were sent a questionnaire every three months to inquire about work-status, additional health costs and doctor visits. The patients who have minimum followup of two years were included.

**Results:**

Of thirty-two patients, 30 met the criterion of the followup period of at least two years. Fourteen patients received nonsurgical therapy, while 16 received surgical treatment. Direct costs of the treatment of nonsurgically treated patients were €10,608 ($12,730). For the operatively treated group, these costs were €18,769 ($22,523). Indirect costs resulted in a total of €219,187 ($263,025) per nonoperatively treated patient. In the operatively treated group, these costs were €66,004 ($79,206).

**Conclusion:**

In the treatment of traumatic thoracolumbar spine fractures, the indirect costs exceed the direct costs by far and make up 95.4% of the total costs for treatment in nonsurgically treated patients and 71.6% of the total costs in the operative group. In view of cost-effectiveness, the operative therapy of traumatic thoracolumbar spine fractures is to be preferred.

Spinal fractures can be an important cause for disabling back pain. Back pain is a major health problem in industrialized countries. The magnitude of the problem is obvious when expressed as incidence and prevalence figures. The annual incidence of back pain in the United States (US) has been reported to be about 5%.^1^ The yearly prevalence of back pain in the US is reported to be 15-20%; and in European countries, 25-40%. The lifetime prevalence in Sweden has been reported to exceed 70%.^2^ Back pain is a frequent cause for visiting a GP or a physical therapist and constitutes a major cause of absence from work and disablement.

The direct costs for the treatment of traumatic thoracolumbar spine fractures are up to two times higher for surgical therapy compared with nonsurgical therapy.^3^,^4^ Because these patients are still in their productive working years and most patients are employed, our hypothesis is that because of the disablement the indirect costs are more important in determining the cost-effectiveness of treatment. As far as we know, there has been no cost-effectiveness study for the treatment of this injury; hence in this prospective randomized study, the costs incurred by nonsurgically and surgically treated patients with a traumatic thoracolumbar spine fracture without neurological involvement were compared.

## MATERIALS AND METHODS

32 patients of traumatic fracture of T10 - L4, AO type A (compression fracture) with no neurological deficit (ASIA/Frankel E) and age 18-60 years from October 1998 until October 2003 were included. Exclusion criteria were AO type A.1.1 fracture, pregnancy, pathologic or osteoporotic fracture, patients with end-stage disease (ASA IV), patients with a history of previous back surgery, patients with a recent psychiatric history, patients using drugs or other illegal substances or patients presenting with any accompanying injury that might interfere with the treatment of the spine fracture or the mobilization scheme after hospital discharge (e.g., lower extremity injuries prohibiting early weight-bearing motion) and any other type of disablement which could interfere with return to work. Patients were randomized between nonsurgical and surgical therapy. All patients were included based on written informed consent. This study was approved by the local ethical committee. Patients were sent a questionnaire every three months to inquire about work-status, additional health costs and doctor visits. Roland Morris Disability Questionnaire-24 (RMDQ-24), VAS Pain and VAS Spine Scores were used to evaluate the patients' well-being. RMDQ-24 is a validated questionnaire to measure self-assessed disability due to back pain. The disability questionnaire was constructed by choosing statements from the Sickness Impact Profile. Patients are given a score of one point for each of the 24 items of the questionnaire that were ticked. A patient's score could thus vary from zero (no disability) to 24 (severe disability).^5^ The VAS Spine Score is developed in Hannover, Germany. The questionnaire is composed of 19 questions which are scored on a 100 millimeter visual analogue scale. The patient's perception of pain and restriction in activities, related to problems of the back, is measured. The score is calculated by taking the average score of all questions and can be any value between zero (severe disability) and 100 (no disability).^6^

The patients who were randomized to be managed nonsurgically were treated with a course of six weeks on a Rotorest bed (KCI, San Antonio, U.S.A.).^7^ Fraxiparine^©^ (nadroparine) 2850 IU was given as an anticoagulant until the patient was discharged. A Jewett hyperextension orthosis was fitted, and the patients were instructed to wear the brace at all times, except when bathing, for three months.^7^ A vigorous physiotherapy scheme was given to improve trunk musculature. The orthosis prevents gross motions of the trunk rather than intervertebral mobility.^8^ Patients were discharged when their pain was in control, and they were self sufficient. Patients were prohibited from engaging in heavy work and sports for three months but were allowed activities of daily living and sedentary work.

The patients who were randomized to receive surgical therapy were managed with a short-segment posterolateral fixation with an AO titanium Universal Spine System [USS (Synthes, Bettlach, Switzerland)].^9^,^10^ Patients were taken to the operation room on a priority rather than an emergent basis. Reduction was indirectly performed with positioning on the operating table. The procedure consisted of a midline incision centered over the fractured vertebra; exposure out of the tips of the transverse processes; placement of the pedicle screws in the level above and in the level below the fractured vertebra. Kyphosis and vertebral body height were corrected through manipulating the pedicle screws. An autogenous bone-graft was harvested from the posterior iliac crest for transpedicular bone grafting.[11] A drill hole to access into the vertebra was made following the central axis of the pedicle. With the aid of an impactor, the central cavity of the vertebra was filled solidly with autogenous bone. A cross-link was used for added stability of the construct.^10^,^12^–^16^ Intra-operative neurologic monitoring was not used.

Preoperative 1500 milligrams of Zinacef^©^ (cefuroxime) was given intravenously as prophylactic antibiotic. Fraxiparine^©^ (nadroparine) 2850 IU was given as an anticoagulant until the patient was discharged. After operation, the patient was mobilized with a Jewett hyperextension orthosis for three months. A vigorous physiotherapy scheme was given to improve trunk musculature. After 9 till 12 months, the USS was removed.

### Cost of illness

The approach most frequently used to estimate the cost of illness (COI) is the human capital approach.[17] According to this approach, the direct costs are estimated on the basis of market prices and the indirect costs by assessing the loss of productivity due to morbidity and premature mortality. This method was used to estimate the direct and indirect costs of the treatment. All costs are presented in Euros (€) and US dollars ($) using an exchange rate of 1.00:1.20.

#### Direct costs:

Direct costs consist primarily of medical costs of diagnosis, treatment, continuing care and rehabilitation. It also includes nonmedical expenditures caused by disease, e.g., travel expenses.^17^ To determine nonmedical expenditures, all patients were sent a questionnaire each and were given three months to register their expenditures.

#### Hospital-care costs:

The hospital-care costs include the costs of clinical care; and additional costs of treatment, diagnostics, paramedical care and operating rooms. The hospital-care costs are divided into outpatient costs and inpatient costs. Since the data concerning medical equipment (i.e., surgical devices, radiological equipment) were not separately recorded, it was not possible to include these costs into this calculation. The following cost categories were used from a former study performed in our clinic.^3^

1. The costs of hospitalization days (normal care, special care and intensive care, including costs of residents/ physicians and staff members, nursing staff, medication, overheads, cleaning, laundry and housing); 2. costs of laboratory tests; 3. costs of blood products; 4. costs of radiology (including X rays and CT scans of the thoracolumbar spine); 4. costs of physical therapy (staff and overheads); 5. costs of operating room, overheads, staff and implant; 6. costs of spinal orthosis and cast and 7. the costs of outpatient visits (including costs of radiology).

#### Medical specialist care costs:

The costs of procedures performed by medical specialists were calculated using the relevant costs for each procedure. The costs incurred were the referring costs for the operation, for anesthesiology and for additional support.

#### General practice care costs:

After discharge from the clinic, part of the care is also delivered by the general physician (e.g., prescriptions for pain-medication). To make an estimation of the costs towards the general physician, patients indicated how often and how many minutes they visited a GP.

#### Indirect costs:

Indirect costs of disease are defined as production losses and related costs to society due to morbidity and mortality.^18^ In case of thoracolumbar spine fractures, these can be the result of absence from work and disablement due to morbidity. There was no mortality rate in this study group; so indirect costs of mortality were not calculated.

The social security system in the Netherlands is based on two laws prescribing the obligation to have insurance for the loss of income due to sickness, injury or disability. Under the Sickness Benefits Act (SBA), workers receive ‘sick’ pay during absence, of a maximum of 52 weeks. If the worker is still unable to work after 52 weeks, the patient is entitled to a disability pension covered by the Disablement Insurance Act (DIA).

#### Costs of absenteeism:

In the present study, costs of absenteeism have been estimated by multiplying the total number of sick days with the mean costs of one sick day. The gross sick pay starts after two qualifying days for the sickness benefit and amounts to 70% of the daily wage. However, due to additional insurances or collective labor agreements, almost every employee receives full wages during absenteeism. We therefore extrapolated the sick pay to 100%. Besides the insurance costs, the Sickness Benefits Act also involves administration costs. In 1999 the total administration costs amounted to 5.5% of the total insurance costs. The administration costs were estimated by adding this proportion to the insurance costs.

#### Costs of disablement:

The total costs of disablement were estimated by multiplying the total days of disability with the mean daily pension in 2000. This was €18,500 ($22,200) per year for men and €12,000 ($14,400) per year for women.

## RESULTS

Of the 32 included patients, two were lost to followup and could not be contacted. 30 patients with a followup of at least two years were included for analysis.

14 patients received nonsurgical therapy and 16 surgical treatment. Mean age was 45.7 years (range 27-59) in the surgically treated patients and 37.3 years (range 18-53) in the nonsurgical group (statistically not significant). The male-to-female ratio was 10:7 in the surgical and 10:5 in the nonsurgical group (not significant). More than 80% of the fractures appeared at the levels Th12 and L1, without a significant difference concerning fracture localization in the two treatment groups. The predominant trauma mechanisms leading to the thoracolumbar fracture were motor vehicle accident and fall from height. At hospital admission, 25 fractures (78%) were classified as type A3, five fractures as type A1 and two fractures as type A2 according to the comprehensive classification. There was no significant difference in distribution of various type A fractures between the two groups. During operation, two type B fractures (distraction type) were identified showing evidence of injuries to the posterior ligamentous complex. Both fractures were included in the study, based on the ‘intention to treat’ principle. In these two specific patients, hardware was not removed. MRI studies were not routinely performed; so the number of unrecognized type B fractures in the nonsurgical group is unknown. The mean Load Sharing Classification in the surgically treated patients was 6.5 (SD 1.12, range 4-8); and in the nonsurgical group, 6.1 (SD 0.96, range 4-9). Considering the LSC scores, there was no significant difference between the two groups.

The estimated pre-injury pain scores as measured by VAS Pain (0 = worst pain imaginable, 100 = no pain at all) averaged 94 mm (range 75-100) for the nonsurgical group and 94 mm (range 78-100) for the surgical group. At final followup, the average scores were 72 mm (15-100) and 87 mm (55-100) for the nonsurgical and surgical groups respectively. The surgically treated patients had significant lesser pains score (*P* = 0.033).

The estimated pre-injury VAS Spine scores (0 = worst pain imaginable, 100 = no pain at all) averaged 94 (74-100) in the nonsurgical group and 87 (61-100) in the surgical group. At final followup, the VAS Spine score was 61 (11-100) for the nonsurgical group versus 81 (45-100) for the surgical group. This difference in score in favor of the surgical treatment was significant (*P* = 0.020).

The Roland Morris Disability Questionnnaire-24 (RMDQ-24) pre-injury functional disability scores (0 = no disability at all, 24 = severe disability), which were estimated on hospital admission, show no significant difference between the two treatment groups: an average of 0.1 (0-2) for the nonsurgical group and 0.6 (0-6) for the surgical group. At final followup, the average score was 8.9 (0-24) for the nonsurgical group and 3.1 (0-14) for the surgical group. The patients receiving nonsurgical treatment were found to have a significantly higher RMDQ-24 score in comparison with the surgical group (*P* = 0.030).

In both the groups, the Pearson's rank correlation test found no correlation between the final amount of local and regional kyphotic deformity and disability according to RMDQ-24 (r = −0.30, *P* = 0.09 for correlation with LSA and r = −0.29, *P* = 0.11 for correlation with RSA), VAS Spine Score (r = 0.22, *P* = 0.23 for LSA; and r = 0.16, *P* = 0.39 for RSA) and VAS pain (r = 0.20, *P* = 0.29 for LSA; and r = 0.17, *P* = 0.38 for RSA).

The surgically treated patients returned to work early and the difference was statistically significant (*P* = 0.04). Five of the 11 (46%) working patients in the nonsurgically treated group returned to their job - three of them to the same job and two did return to a job which was less demanding. The average time for returning to work for nonsurgical patients was 14 months (range 6-33). Nine of the 11 (82%) working patients in the surgical group returned to their previous job; none of them changed to a physically less demanding job. Average returning time in the operatively treated group was 7 months (1-18). No significant difference between the nonsurgical and surgical groups could be reached because of the spread in time to return to work from one till 33 months.

### Direct costs

#### Hospital-care costs:

Direct costs of the treatment of nonsurgically treated patients were €9,900 ($11,880), which were €18,300 ($21,960) for the surgically treated group.^3^ Probably the total costs of treatment will be higher because of under-registration and not taking into account the costs of supporting faculties like administration and kitchen and costs for water and electricity.

General practice care costs: Costs of a GP are €47 ($56) per hour. Patients who were treated nonoperatively visited the GP for 500 minutes. Total costs were €392 ($470), resulting in €28 ($34) per patient. Surgically treated patients spent 214 minutes at their GP. Total costs were €169 ($203), resulting in €11 ($13) per patient.

#### Private expenditures of the patient:

Examples of expenditures made were clothing, education for a job change, physiotherapy and transportation costs. In the nonsurgically treated group, these expenditures totaled €9,525 ($11,430) or €680 ($816) per patient. The surgical group spent €7,322 ($8,786), resulting in €458 ($550) per patient.

### Indirect costs

#### Costs of absenteeism:

In the nonoperatively treated group, 11 patients did have a regular job. The total period of absence of these 11 patients was 115 months. This resulted in total costs of €120,500 ($144,600) or €8,607 ($10,329) per nonsurgically treated patient. Twelve patients in the surgical group had a regular job. The total period of absence was 78 months. This resulted in total costs of €88,400 ($106,080) or €5,525 ($6,630) per operatively treated patient. In Table 1, 5.5% administration costs are added.

**Table 1:** Shows costs incurred per patient in surgical and nonsurgical group for treatment of thoracolumbar trauma

Costs per patient	Nonsurgical	Surgical
Direct		
Hospital costs	€9,900	€18,300
	($11,880)	($21,960)
Costs for GP	€28	€11
	($34)	($13)
Private expenditures	€680	€458
	($816)	($550
Subtotal	€10,608	€18,769
	($12,730)	($22,523
Indirect		
Costs of absenteeism	€8,607	€5,525
	($10,329)	($6,630
Administration costs	€473	€304
	($568)	($365)
Costs of disablement	€210,107	€41,406
	($252,128)	($49,688)
Subtotal	€219,187	€47,235
	($263,025)	($56,683)
Total	€229,795	€66,004
	($275,755)	($79,206)

#### Costs of disablement:

Because of our strict inclusion and exclusion criteria (e.g., no additional injury, no disablement which could interfere with therapy or return to work), the disablement in our group was caused by the thoracolumbar spine fracture in all cases. Through the questionnaire, we could verify that the disablement was caused by residual back pain or was treatment related. In the nonoperatively treated group of patients, six were disabled. These were all men with a mean age of 39 years (range 18-58). One hundred fifty-nine years have to be compensated. This results in a total compensation of €2,941,500 ($3,529,800), or €210,107 ($252,128) per nonoperatively treated patient. In the surgical group, there were two disabled patients, one man aged 48 and one woman aged 36. Thus a total of 46 years have to be compensated. This results in a total compensation of €662,500 ($795,000), or €41,406 ($49,688) per operatively treated patient.

## DISCUSSION

This is the first study which studies prospectively the costs that are made in the treatment of traumatic thoracolumbar spine fractures and in which direct and indirect costs are calculated. We also took this opportunity to compare the costs of nonoperative and operative treatment to see which therapy is more cost-effective. Cost-effectiveness can be used as a rational way of deciding how to get the best health improvement from limited resources - which is a reasonable, even essential, concern.^19^

Two studies reported direct costs of treatment of thoracolumbar spine fractures. The results for the direct costs reported in one of these studies are used in this report.^3^ The estimated direct costs in the study by Hitchon *et al.* were higher than the costs in this study - $24,600 versus $12,730 for nonsurgical treatment and $45,300 versus $22,523 for surgically treated patients.^4^ Hospitalization days for patients treated nonsurgically were about the same in both the studies. In the study by Hitchon, operatively treated patients had on an average seven more hospitalization days. Unit prices may also have been higher in the study by Hitchon, explaining the difference in costs.^3^,^4^

Our hypothesis that indirect costs are a more important factor than the direct costs in the treatment of thoracolumbar spine fractures was confirmed. The indirect costs exceed the direct costs by far and make up 95.4% of the total costs for treatment in nonsurgically treated patients and 71.6% of the total costs in the surgical group. The costs of disablement are an important factor. When a patient aged 18 years becomes disabled and is unfit for work, 47 years have to be compensated. This stresses the importance of the indirect costs in judging cost-effectiveness. In comparative studies, these indirect costs are never addressed, which makes a good cost-effectiveness study impossible, resulting in charges for operative therapy to be as much as four times the charges for conservative therapy.^19^ This study shows that indirect costs form the major proportion of costs in both treatment options, and nonsurgical treatment is 3.5 times more expensive than surgical treatment when one takes into account both direct and indirect costs. Although the differences in health and insurance systems between the United States, India and the Netherlands make comparison difficult, indirect costs are the most important factor in judging the cost-effectiveness of the treatment of traumatic thoracolumbar spine fractures.

It seems justified to state that in view of cost-effectiveness, the operative surgical therapy of traumatic thoracolumbar type A (excluding A.1.1) spine fractures is to be preferred because of the lower indirect costs.
